# Identification of bacterial communities in extreme sites of Pakistan using high throughput barcoded amplicon sequencing

**DOI:** 10.3897/BDJ.9.e68929

**Published:** 2021-10-20

**Authors:** Anila Fariq, Azra Yasmin, John C Blazier, Sammyia Jannat

**Affiliations:** 1 Department of Biotechnology, Fatima Jinnah Women University, Rawalpindi, Pakistan Department of Biotechnology, Fatima Jinnah Women University Rawalpindi Pakistan; 2 Department of Biotechnology, University of Kotli, AJK, Kotli, Pakistan Department of Biotechnology, University of Kotli, AJK Kotli Pakistan; 3 Texas A&M Institute of Genome Sciences and SocietyTexas A&M University,, College Station, Texas, United States of America Texas A&M Institute of Genome Sciences and SocietyTexas A&M University, College Station, Texas United States of America

**Keywords:** culture independent, barcoded amplicon sequencing, operational taxonomic units, poly-extremophilic

## Abstract

Microorganisms thrive nearly everywhere including extreme environments where few other forms of life can exist. Geochemistry of extreme sites plays a major role in shaping these microbial communities and microbes thriving in such harsh conditions are untapped sources of novel biomolecules. To understand the structure and composition of such microbial communities, culture-independent bacterial diversity was characterised for two extreme sites in Pakistan, Khewra salt range and Murtazaabad hot spring. Barcoded amplicon sequencing technique was used to study the microbial communities. Physicochemical analysis of these sites was also conducted to study the dynamics of microbial communities under stressed conditions. Metagenomic sequencing of salt range soil samples yielded of 40,433 16S rRNA sequences, while hot spring sediments produced 76,449 16S rRNA sequence reads. Proteobacteria were predominant in saline soil while Firmicutes were most abundant in hot spring sediment. The taxonomic analysis of saline samples revealed 914 operational taxonomic units (OTUs) while that of hot spring sequences were clustered into 726 distinct OTUs. OTUs from genus *Alkalibacillus* were most abundant in hot spring sediments, whereas *Haloarcula* were more prevalent in saline soil. Some unidentified sequences were also present at each taxonomic level. Multivariate analysis indicated that electrical conductivity and pH are the major environmental factors involved in modelling microbial communities. This study revealed a poly-extremophilic microbial community in the Murtazaabad hot spring and characterised the unexplored halophilic microbial diversity of saline soil of Pakistan.

## Introduction

Many prokaryotes reside in extreme environments in which some chemical or physical environmental parameters vary considerably from the normal habitats that support life. Such organisms are called extremophiles, flourishing in habitats which are hostile for other living organisms. Isolation and characterisation of extremophilic prokaryotes in recent years revealed their metabolic potential (Jiang et al. 2006).

The development of molecular tools and the ability to isolate microbes in laboratory culture have revolutionised knowledge of bacterial diversity, which greatly exceeds that found in eukaryotes. There is currently great interest in mining the genetic resources of prokaryotic cells to be used in biotechnology and related areas (Madigan 2000, Rampelotto 2013). It is widely known that culture-dependent methods reflected only a small part of real diversity in natural environments and that culture-independent molecular techniques are vital tools for studying the evolution and diversity of microbes and characterising microbial communities. Only 0.1–10% microorganisms of the total biosphere have been cultured so far. Therefore, culture-independent molecular approaches provide new perspectives for studying the composition and dynamics of microbial communities inhabiting soil (Horneck et al. 2010, Tahir et al. 2015).

Modern metagenomic studies, like 16S rRNA sequencing, accurately characterise microbial diversity. However, many types of locale are still un-sampled and extensive effort is required to determine the patterns of microbial ecology and evolution in such extreme environments. Nowadays, these communities have gained more attention in applied research both due to their biotechnological potential and to comprehend the evolution of biomolecules from their analogues found in other organisms (Sahay et al. 2017).

A study reported the microbial diversity of three hot springs from Neuquén, Argentina, by using molecular based high-throughput amplicon sequencing technique. This study demonstrated metabolic profiling in the acidic and the circum-neutral samples as the former were dominated by chemolithotrophs, while the latter were dominated by chemoheterotrophs. The research also described that microbial communities were shaped by complex factors other than pH and temperature (Massello et al. 2020). In another study, Mashzhan et al. 2021 assessed the diversity of the microbial community in the Zharkent geothermal hot spring in the south-eastern region of Kazakhstan, using culture-dependent and -independent approaches. They reported that spring water yielded 11,061,725 high-quality sequence reads and more than 99.97% of the total prokaryotic abundance comprised of bacteria, with Archaea contributing only a small constituent of the community. Similarly, in another study, fluorescence in situ hybridisation, used to determine prokaryotes diversity in Urmia Salt Lake, revealed that the proportion of bacteria and archaea ranged between 36.1-55% and 48.5-55.5%, respectively (Jookar Kashi et al. 2021). Uritskiy et al. (2020) described extremophile microbial communities inhabiting salt rocks in the Atacama Desert, Chile, as a model ecosystem to study microbiome heterogeneity because of their diverse taxonomic composition and the spatial segregation of distinct nodule.

Pakistan is situated in the sub-continent along the junctions of the tectonic plates and is rich in geothermal resources. The present geological structure of Pakistan was formed by the major tectonic elements in the Cenozoic and Mesozoic era. The distribution of hot springs reflects the movements of tectonic plates. These hot springs are distributed in Chilas and Hunza along the plunge of main mantle and Karakoram. Temperature of these hot springs may reach 96°C (Javed et al. 2012). As the most extreme hypersaline site of Pakistan, the Khewra salt range represents a totally different type of extremophile habitat. The salt mines are largest in terms of area and one of the largest producer of rock salt in the world. Comprehensive microbial diversity of this extreme site is yet unexplored.

The present study aims to investigate the composition of bacterial communities of extreme sites of Pakistan including Murtazabad hot spring and Khewra salt range by using Bacterial tag-encoded FLX amplicon pyrosequencing (bTEFAP) (Mashzhan et al. 2021).

## Materials and methods

### Sample collection

The saline soil sample was collected from Khewra salt range at the latitude and longitude of 32ºN, 73ºE, while the sediment sample was collected from the hot spring of Murtazaabad, located in Gilgit Baltistan at 35ºN and 76º E, respectively. Six soil samples from each site were collected at a depth of 0 to 5 cm by metallic tubes with a diameter of 5 cm, mixed evenly and placed in sterile zipper plastic storage bags.

### Physicochemical analysis

To determine the electrical conductivity and pH of samples, aqueous solutions of samples were prepared in the ratio of 1:10 and electrical conductivity was measured by EC meter, while pH was measured by pH meter. For metal analysis, soil was digested by the acid digestion method (Das and Ting 2017). The concentration of different metals was determined through flame atomic absorption spectrophotometer (Model 220 Spectra AA Varian).

### Barcoded amplicon sequencing

Total soil DNA was extracted with QIAGEN PowerSoil kit by following standard protocols. Barcoded amplicon sequencing (bTEFAP®) was performed by MR DNA as described by Dowd et al. (2008) to characterise the environmental microbiome. For identification of 16S rRNA genes, PCR primers 515/806 were used with the barcode on the forward primer. A 30 cycle PCR was set up for DNA amplification under the following conditions: Denatuartion at 94°C for 3 minutes, annealing at 94°C for 30 seconds (28 cycles), 53°C for 40 seconds, 72°C for 1 minute and finally extension for 72°C for 5 minutes. About 2% agarose gel was used to visualise PCR products. All samples were pooled together and purified. The DNA library was prepared from purified PCR products following Illumina TruSeq DNA library preparation protocols. Sequencing was done at MR DNA (www.mrdnalab.com, Shallowater, TX, USA) on a MiSeq platform following the manufacturer's protocol. This is an Illumina's next generation sequencing instrument and one of the smallest benchtop sequencers used to perform onboard functions like cluster generation, amplification, genomic DNA sequencing and data analysis in a single run (Liu et al. 2012).

### Data analysis

The QIIME data analysis package was used for 16S rRNA data analysis (Caporaso et al. 2010). Open OTU picking was performed in QIIME using a similarity threshold of 0.97 and the Greengenes 16S database. Low-confidence OTUs with an abundance of < 0.01% in the dataset were removed with the 3rd party script remove_low_confidence_otus.py from the Microbiome Helper package (Comeau et al. 2017). Taxonomic plots were generated and statistical tests were performed in QIIME. Canonical Correspondence analysis was performed in PAST software to determine the relationships between biological assemblages and their environment.

## Results

Geochemistry of the saline sample showed neutral pH and very high electrical conductivity (19 mS/cm) reflecting the hypersaline nature of the sample. The hot spring sediment sample exhibited alkaline pH and conductivity of 2.17 mS/cm. Both samples contained variable concentrations of different heavy metals i.e. Pb, Ni, Cu, Zn, Cd and large concentrations of Ca, K and Na (Table 1).

**Figure 1.**
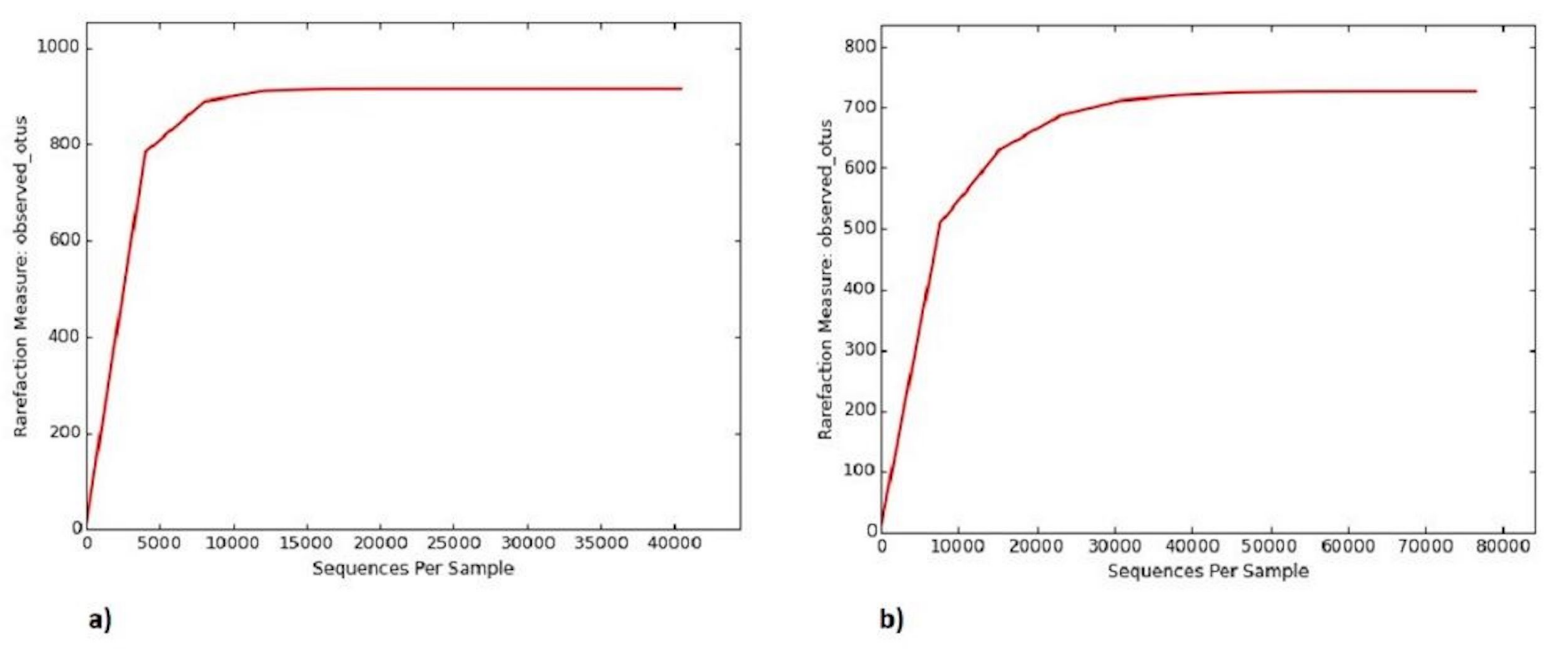
Rarefaction curves estimating the diversity in **a**) saline soil; **b**) hot spring sediment.

G+C content of the saline sample was about 56% and that of the sediment sample was 56.5%. About 40,433 high quality 16S rRNA sequences obtained from saline sample and 76,449 from sediment sample were clustered into 914 and 726 operational taxonomic units (OTUs), respectively after filtering low-confidence sequences with fewer than three counts. Rarefaction curves estimated the diversity captured in each sample (Fig. 1a-b). Rarefaction curves were used for the determination of microbial diversity coverage. This is a qualitative method widely used to estimate coverage and relies on the curve of rarefied counts of operational taxonomic units. If the sample is close to saturation, the curve should be like a plateau (Rodriguez-R and Konstantinidis 2014). Environmental heterogeneity of the samples directly influences these rarefaction curves. It is one of the major factors related to microbial diversity assuming that environments with higher environmental heterogeneity host more species as they provide diverse available habitats (Rocchini et al. 2012).

**Figure 2.**
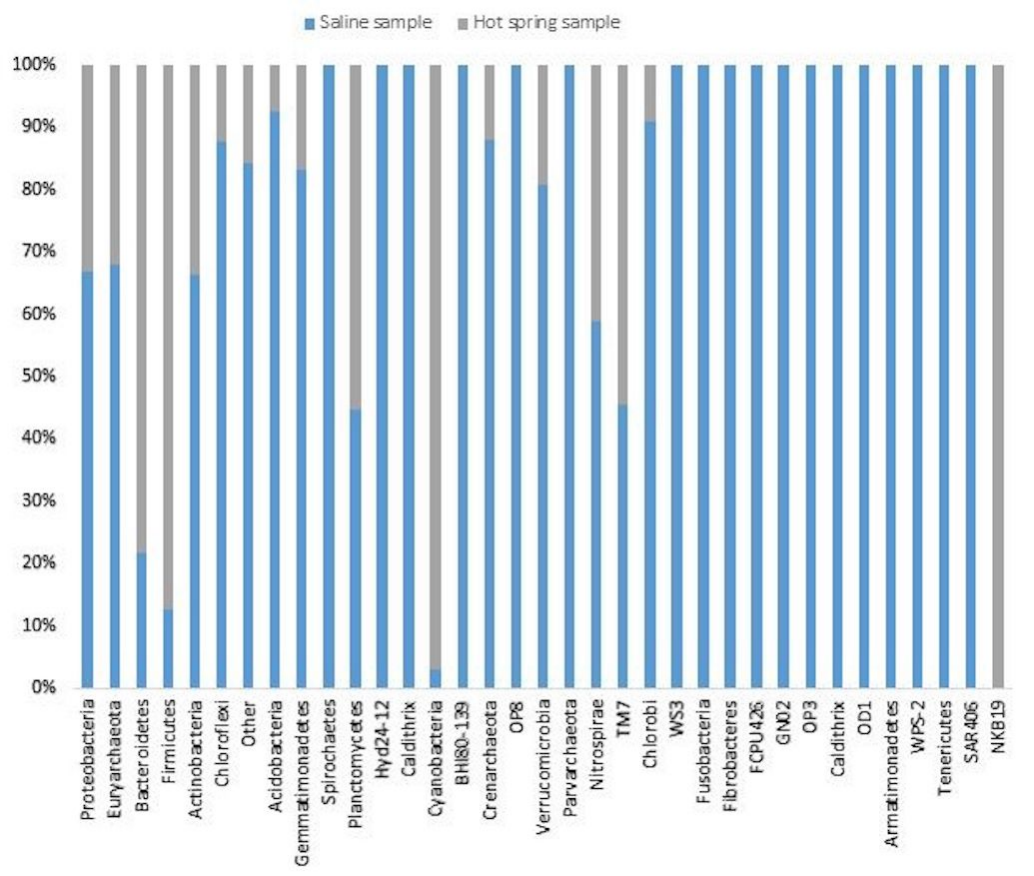
Percentage abundance of different phyla in saline and hot spring samples.

In the saline sample, a total of 35 distinct prokaryotic phyla were detected by metagenomic analysis, out of which Proteobacteria account for 46.20% of the total biodiversity. Amongst Proteobacteria, Gammaproteobacteria was the most prevalent class of bacteria. Deltaproteobacteria was comparatively less abundant. Alphaproteobacteria and Betaproteobacteria were poorly represented. Euryarchaeota was the second most abundant phylum and contributed to 25.38% of diversity. In archaea, Halobacteria was the most predominant class, as expected in an extreme halophilic community. Other relatively less abundant phyla found in hypersaline soil included Actinobactria (3.53%), Bacteroidetes (4.24%), Firmicutes (4.14%), Acidobacteria (2.24%), Chloroflexi (2.85%), Gemmatimonadetes (2.10%), Spirochetes (1.64%) and Cyanobacteria (0.41%). About 2.47% of the total phyla were not assigned to any category and classified as “other”. In the hot spring sample, 19 different phyla were identified. Firmicutes, Proteobacteria, Bacteroidetes, Cyanobacteria and Euryarchaeota constituted the major components with 29.14, 22.81, 15.29, 13.77 and 12.08%, respectively of total bacterial population. Other relatively less dominant phyla include Actinobacteria, Planctomycetes, Gemmatimonadetes, Chloroflexi, Acidobacteria, Crenarchaeota and Nitrospirae (Fig. 2).

**Figure 3.**
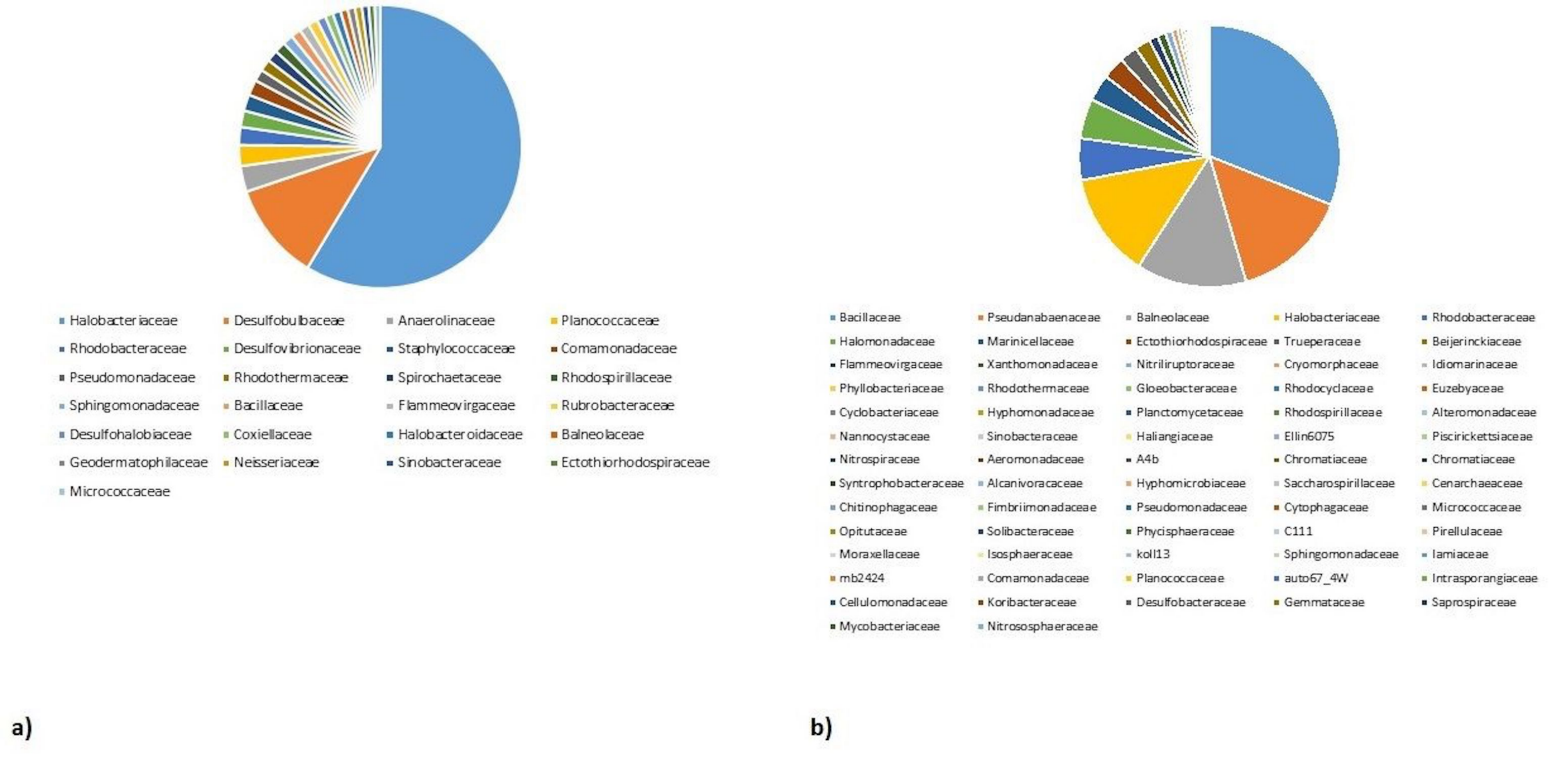
Percentage abundance of families identified in **a**) saline soil; **b**) hot spring sediment.

At the family level in the saline sample, Halobacteriaceae is the most predominant family displaying 25.19% of the total population. Other families identified in the metagenomic data (Fig. 3a) include Desulfobulbaceae, Anaerolinaceae, Planococcaceae, Rhodobacteraceae, Desulfovibrionaceae, Staphylococcaceae, Pseudomonadaceae and Rhodothermaceae. In the hot spring metagenomic data, about 109 distinct families were represented, out of which Bacillaceae was the most dominant, accounting for 29.09% of the total bacterial population. Pseudanabaenaceae, Balneolaceae, Halobacteriaceae and Rhodobacteraceae comprised 13.51%, 12.80%, 4.85% and 4.59% of the population, respectively (Fig. 3b).

**Figure 4.**
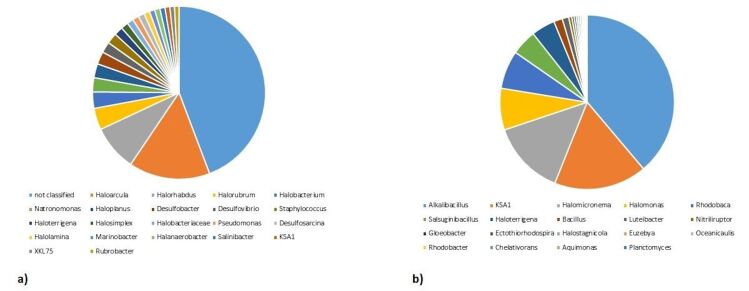
Percentage abundance of genera identified in **a**) saline soil; **b**) hot spring sediment.

In the saline sample, at the genus level, *Haloarcula*, *Halorhabdus*, *Halorubrum*, *Halobacterium*, *Haloplanus*, *Natronomonas*, *Desulfobacter*, *Haloterrigena*, *Desulfovibrio*, *Desulfococcus*, *Staphylococcus*, *Pseudomomnas*, *Halolamina*, *Halosimplex*, *Desulfoarcina*, *Peptococcus*, *Marinobacter*, *Rubrobacter* and *Salinibacter* were identified (Fig. 4a). By contrast, in the hot springs sediment sample, *Alkalibacillus*, *Halomicronema*, *Halomonas*, *Rhodobaca*, *Salsuginibacillus* and *Haloterrigena* were identified (Fig. 4b).

**Figure 5.**
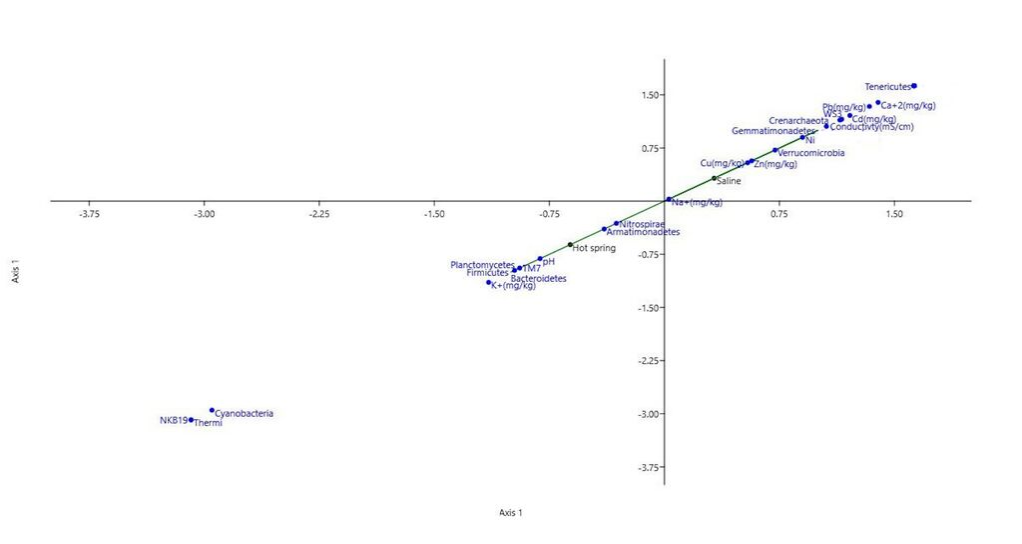
Relationship between bacterial diversity and different physicochemical parameters of the samples using Canonical Correspondence Analysis (CCA) plot.

Canonical Correspondence Analysis was performed to study the effect of physicochemical parameters on bacterial communities. In the saline sample, species richness and abundance was attributed to the electrical conductivity, Na^+^, Ca^+2^ and other heavy metal concentrations. In the hot springs sediment sample, pH and K^+^ concentration are crucial in shaping bacterial community (Fig. 5).

## Discussion

Microbial community structure and function can be efficiently demonstrated by their microbial diversity (Yousuf et al. 2012). Additionally, geochemical characteristics of the environment define the microbial community of a particular area. Temperature of the hot water spring of Murtazabad ranged from 39°C to 75°C. The physicochemical properties of sediment revealed an alkaline nature of sediment which mimics overall geochemistry of nearby soils. Geochemistry of the saline sample also indicated very high values of electrical conductivity and metals. These high values are attributed to the hypersaline environment. The present study demonstrates that environmental variables play a significant role in determining bacterial communities. It is generally assumed that salinity is an important factor in shaping microbial communities. Canfora et al. (2014) observed a strong correlation between soil properties and the occurrence of bacterial phyla. They demonstrated the role of salt concentrations in determining bacterial diversity of saline soil and their effect on bacterial community structure.

Bacterial diversity of hot spring sediments detected an abundance of Firmicutes and *Proteobacteria*. Our results are consistent with Chan et al. (2016), who determined culture-independent diversity of thermophiles in Malaysian hot springs. Their study found that the community was dominated by Firmicutes and *Proteobacteria*, together comprising 57% of the bacterial population. Chaudhuri et al. (2017) also reported that Firmicutes dominated in microbial communities of hot springs of West Bengal, India. Abundance of Firmicutes is also attributed to the lack of nutrients availability (Kambura et al. 2016). Surprisingly, the most dominant genus, identified in the present study, was *Alkalibacillus* which indicated the influence of pH on bacterial diversity as was also shown in Canonical Correspondence Analysis. Interestingly, our results varied from a previous study on diversity of hot springs in Pakistan where members of *Chloroflexi* were most dominant (Amin et al. 2017). Many unidentified lineages were also detected in the present study at different taxonomic levels which might be the consequence in the complex physiology of extremophilic communities.

Prokaryotic halophiles are ubiquitous in nature and have been studied using both culture-based techniques and culture-independent sequence-based approaches. Sequence-based environmental metagenomic studies are rapidly increasing the existing knowledge of non-cultivable microbial communities, such as halophilic bacteria and archaea (DasSarma and DasSarma 2017). Metagenomic analysis of hypersaline soil of Khewra mine revealed a diverse microbial community that comprised of around 305 different genera. Proteobacteria represent the most dominant phylum of the community. It is an important group of microorganisms in terms of its evolutionary, geological and environmental significance. All Proteobacteria are characteristically Gram-negative, facultative or obligate anaerobes having gas vesicles, flagella or can move by gliding. They are chemoautotrophs, chemoorganotrophs or phototrophs of medical, industrial and agricultural significance (Marin 2011). Members of this phylum possess vast physiological, morphological and metabolic diversity and play significant roles in global carbon, nitrogen and sulphur cycling. Nonetheless, the majority of soil Proteobacteria remain unexplored. Irrespective of the sequencing technique used, metagenome studies of soil reflect Proteobacteria as the most abundant soil phylum (Spain et al. 2009).

*Euryarchaeota* was second most abundant phylum in saline sample. The archaeal domain is a third line of evolutionary lineage varying from bacteria and eukarya. Most of studies limit archaea to extreme environmental conditions (Chaban et al. 2006). Halophilic archaea are physiologically, metabolically and phylogenetically diverse group of microbes, categorised on the basis of their obligate halophilic regime, characteristic red colouration and aerobic heterotrophic metabolism (Youssef et al. 2012). Canfora et al. (2014) reported microbial diversity of natural saline soil located in Sicily (Italy) with dominant taxa Proteobacteria, Actinobacteria, Acidobacteria, Verrucomicrobia, Firmicutes and less common Bacteroidetes, Chloroflexi, Chlorobi and Gemmatomonadates. In another study, Yang et al. (2016) demonstrated the effect of salinity on shaping the microbial community structure of surface sediments of the Qinghai-Tibetan Lakes. In their study, Proteobacteria was the most abundant phylum and Euryarchaeota, Acidobacteria, Actinobacteria, Bacteroidetes, Chloroflexi, Cyanobacteria, Firmicutes, Gemmatimonadetes, Planctomycete, Proteobacteria, Thermi and Verrucomicrobia were present in lower proportions. These studies corroborated with the present study reflecting typical communities of hypersaline niches.

Predominant genera, identified in the present study, include *Haloarcula*, *Halorhabdus*, *Halorubrum*, *Alkalibacillus*, *Halomicronema*, *Halomonas*, *Rhodobaca*, *Salsuginibacillus* and *Haloterrigena*. Members of the genus *Haloarcula* are Gram-negative, rods or pleomorphic archaea commonly found in hypersaline environments, such as saline soils, salt samples and salt lakes, salterns. They are usually pigmented, neutrophils and extremely halophilic i.e. grow at 1.7–5.2 M sodium chloride (NaCl). Nine species of the genus, identified so far, include *H.vallismortis*, *H.marismortui*, *H.amylolytica*, *H.tradensis*, *H.hispanica*, *H.japonica*, *H.salaria*, *H.quadrata* and *H.argentinensis* (Ventosa et al. 2015). Members of genus *Halorhabdus* are extremely pleomorphic, Gram‐negative, red or non-pigmented, facultative anaerobic or aerobic chemoorganotrophs. *Halorhabdus* species are extremely halophilic, thermos-tolerant i.e. grow in the range of 15–57.5°C and cells lyse in water. Species of the genus *Halorhabdus* i.e. *Hrd.utahensis*, *Hrd.tiamatea* and *Hrd.rudnickae*, have been isolated from hypersaline lakes, deep‐sea brines and salt mine boreholes, respectively (Antunes et al. 2015). *Halorubrum* is the largest genus of class Halobacteria and contains approximately 36 species, including *Halobacteriumsaccharovorum*, *Halobacteriumtrapanicum*, *Halobacteriumlacusprofundi* and *Halobacteriumsodomense*. Species of this genus are aerobic, phenotypically diverse, chemoorganotrophic and obligate halophiles with optimum growth range at 1.0–5.2M NaCl concentration. Different cultivation-based and molecular-based techniques revealed their abundance in hypersaline environments, like hypersaline soda lakes, marine salterns saline soils, salt fermented seafood and salt lakes (de la Haba et al. 2018). Species of the genus *Alkalibacillus* have been identified from salt-lake, alkaline and highly saline mud, water of a mineral pool and non-saline surface soil (Tian et al. 2007). Members of genus *Halomicronema* are moderately halophilic and moderately thermophilic cyanobacteria. The four strains of this genus have been identified with very thin trichomes from benthic microbial mats with growth range at 12-15% (w/v) salinity and 45-50°C temperature (Abed et al. 2002).

### Conclusion

The present study concluded that extreme sites of Pakistan are rich in prokaryotic diversity. Major phyla identified in hot spring samples were poly-extremophiles and have been adapted to more than one extreme conditions. Diversity analysis of saline metagenomes showed abundance of Proteobacteria as a major phylum of halophilic community. Moreover, environmental factors are playing key roles in shaping extremophilic microbial communities. Overall, this study reflected the both culturable and non-culturable prokaryotic diversity of unexplored extreme habitats of Pakistan which can be exploited further for the discovery of novel biomolecules having industrial significance.

## Data Archiving Statement

The original sequencing output files have been deposited in the Sequence Read Archive (SRA) service of the National Centre for Biotechnology Information (NCBI) database under the accession numbers SAMN08026743 and SAMN08026744, respectively.

## Figures and Tables

**Table 1.** Physicochemical properties of samples.

**Parameters**	**Saline soil**	**Hot spring sediment**
**Temperature (°C)**	28	50
**pH**	7.28	7.8
**Conductivty (mS/cm)**	19	2.17
**Pb (mg/kg)**	182.5	12.015
**Ni (mg/kg)**	113.5	20.7
**Cu (mg/kg)**	108.83	32.56
**Zn (mg/kg)**	72.8	21.1
**Cd (mg/kg)**	28.35	2.76
**Na(mg/kg)**	10816	5550
**Ca (mg/kg)**	11475	600
**K (mg/kg)**	6400	9150

## Hosting institution

Department of Biotechnology. Fatima Jinnah Women University, Rawalpindi, Pakistan
